# SG-APSIC1159: Control of hospital-acquired carbapenemase-producing carbapenem-resistant Enterobacteriaceae colonization: A descriptive study

**DOI:** 10.1017/ash.2023.82

**Published:** 2023-03-16

**Authors:** Tuodi Wu, Li Jie, Maria Theresa Mapalo Muyot Cabahug, Philomena Liew, Chai Hairu, Suhailah Binte Nasir, Harminder Kaur, An Rongyan, Foo Shi Yun, Tan Seow Yen, Tan Thean Yen, Tan Si Huei

**Affiliations:** Changi General Hospital, Singapore; Changi General Hospital, Singapore; Changi General Hospital, Singapore; Changi General Hospital, Singapore; Changi General Hospital, Singapore; Changi General Hospital, Singapore; Changi General Hospital, Singapore; Changi General Hospital, Singapore; Changi General Hospital, Singapore; Changi General Hospital, Singapore; Changi General Hospital, Singapore; Changi General Hospital, Singapore

## Abstract

**Objectives:** Carbapenemase-producing carbapenem-resistant Enterobacteriaceae (CP-CRE) are nosocomial pathogens, and control of CP-CRE transmission is one of the most important infection control issues healthcare organizations face today. Increasing colonization acquisition and clinical infections of CP-CRE occurred in our institution in 2019. In this observational study, we monitored CP-CRE acquisition following implementation of multimodal control measures, and we describe the impact of this intervention on clinical infections. **Methods:** Increased hospital-acquired CP-CRE colonization and clinical infections were observed in early 2019. Increased CP-CRE surveillance was implemented to include CP-CRE contacts, patients with lengths of stay >7 days, patients with a recent history of hospitalization in other hospitals, and renal dialysis patients. The following interventions were also implemented**:** (1) isolation or placing CP-CRE patients in cohorts in a designated multidrug-resistant organism (MDRO) ward; (2) emphasis on hand hygiene and contact precautions; (3) mandatory use of gown and gloves for predefined ‘high-risk’ nursing activities, including diaper changing, toilet assistance, wound dressing, and handling urine or stool; (4) enhanced environmental and equipment cleaning; (5) regular audit and feedback regarding compliance; and (6) weekly feedback on ward-level CP-CRE acquisition. CP-CRE colonization cases and clinical infections were tracked by infection prevention and control nurses. **Results:** The hospital-acquired CP-CRE colonization rate was 4.39 per 10,000 patient days in 2019; it decreased slightly to 3.61 in 2020 and remained steady at 3.77 in 2021. The predominant CP-CRE genes were NDM, OXA-48–like, and KPC. There were 12 hospital-acquired CP-CRE infections in 2019, a rate of 0.37 per 10,000 patient days. This incidence decreased to 6 infections in 2020 and 3 infections in 2021, with corresponding infection rates of 0.19 and 0.09 per 10,000 patient days, respectively. **Conclusions:** Control of CP-CRE remains extremely challenging in hospitals with multibed open wards. A bundle approach to infection control showed a gradual reduction in CP-CRE cases, with a significant impact on the prevention of clinical infections.

